# A Single Supratherapeutic Dose of Atogepant Does Not Affect Cardiac Repolarization in Healthy Adults: Results From a Randomized, Single‐Dose, Phase 1 Crossover Trial

**DOI:** 10.1002/cpdd.940

**Published:** 2021-05-04

**Authors:** Ramesh Boinpally, Brian McNamee, Li Yao, Matthew Butler, Danielle McGeeney, Lisa Borbridge, Antonia Periclou

**Affiliations:** ^1^ AbbVie Madison New Jersey USA; ^2^ AbbVie Marlow UK; ^3^ AbbVie Irvine California USA

**Keywords:** atogepant, calcitonin gene–related peptide receptor antagonist, cardiac repolarization, migraine disorders, thorough QT study

## Abstract

Atogepant is a selective, oral calcitonin gene–related peptide receptor antagonist in development for preventive treatment of migraine. This randomized, double‐blind, phase 1 crossover study evaluated the cardiac repolarization effect of a single supratherapeutic (300 mg) atogepant dose vs placebo in healthy adults. Moxifloxacin 400 mg was the open‐label active control. The primary end point was a change from baseline in Fridericia‐corrected QT intervals (ΔQTcF). Sixty participants were randomized to atogepant 300 mg, placebo, and moxifloxacin; 59 (98.3%) completed all interventions. Assay sensitivity was confirmed: lower 90% confidence interval limit for QTcF interval change from baseline (ΔΔQTcF) for moxifloxacin was >5 millisecond vs placebo at prespecified 2‐, 3‐, and 4‐hour time points. Following single‐dose atogepant 300 mg, mean atogepant ΔΔQTcF and upper 90% confidence interval limits were lower than the 10‐millisecond threshold at all time points. Atogepant mean peak plasma concentration was 3197 ng/mL, area under the concentration‐time curve from time 0 to time t was 16 640 ng • h/mL, area under the concentration‐time curve from time 0 to 24 hours was 16 607 ng • h/mL, and median time to peak plasma concentration was 2.1 hours. The incidence of adverse events was low; no serious adverse events or elevations of liver enzymes were reported. Overall, a single supratherapeutic dose of atogepant was safe and did not impact cardiac repolarization in healthy participants.

Migraine is a highly prevalent, chronic neurological disease that imposes substantial burden on individuals and society.^1^, ^2^ Symptoms are often disabling and have a negative impact on many aspects of life, including work productivity, quality of life, and finances.^3^, ^4^, ^5^ New safe and effective treatments are needed to help prevent migraine.

Calcitonin gene–related peptide (CGRP) is a potent vasodilatory neurotransmitter that is believed to play a key role in the pathophysiology of migraine.^6^, ^7^ Medications that target CGRP or its receptors include the small‐molecule oral CGRP receptor antagonists (gepants), such as ubrogepant and rimegepant, which are approved for the acute treatment of migraine attacks, and monoclonal antibodies that block the CGRP pathway and are approved for migraine prevention.^7^ Gepants could provide a valuable treatment option for people who may prefer an oral route of administration for migraine prevention. Additionally, gepants can meet the acute treatment needs of individuals with cardiovascular comorbidities and those with medication overuse.^8^ Because the gepants lack vasoconstrictive properties, they are safe for individuals with migraine who cannot use triptans.^9^

Atogepant is a potent, selective, competitive, oral CGRP receptor antagonist that is in development for preventive treatment of migraine (ClinicalTrials.gov: NCT03700320, NCT03777059, NCT03855137, NCT03939312). Atogepant is rapidly absorbed upon oral administration with a median time to peak plasma concentration of 1 to 2 hours and has a terminal elimination half‐life of ≈11 hours, with no evidence of significant accumulation, with 10‐day (once‐daily) accumulation ratios for area under the concentration‐time curve (AUC) from time 0 to 24 hours and peak plasma concentration (C_max_) between 0.77 and 1.07. Atogepant exhibits dose proportional pharmacokinetics (PK) through 300 mg, the highest dose tested. Atogepant exhibits weak activity on L‐type calcium channels and human ether‐à‐go‐go–related gene potassium channels, with a half maximal inhibitory concentration of 22.5 and 48.2 μm, respectively. Oxidative metabolism of atogepant occurs predominantly via cytochrome P450 (CYP) 3A4 with a minor contribution of CYP2D6. Atogepant is not an inhibitor of CYP1A2 or CYP3A4, and displayed only weak inhibition of CYP2B6, CYP2C8, CYP2C9, CYP2D6, and CYP2C19. Atogepant is not a potent inhibitor of monoamine oxidase A or uridine 5’‐diphospho‐glucuronosyltransferase 1 family, polypeptide A1. Atogepant is not expected to have clinically significant drug‐drug interactions with compounds metabolized by CYP3A4.

The efficacy and safety of atogepant for the preventive treatment of migraine has been demonstrated in a 12‐week, randomized, placebo‐controlled, phase 2b/3 clinical trial, where atogepant had a favorable safety profile with demonstrated efficacy at once‐daily doses of atogepant 10, 30, and 60 mg, and twice‐daily doses of 30 and 60 mg compared with placebo.^10^ An additional 12‐week, randomized, placebo‐controlled, phase 3 trial found significant efficacy with once‐daily doses of atogepant 10, 30, and 60 mg.^11^

The objective of this study was to assess the impact of a single supratherapeutic (300 mg) dose of atogepant on the QT interval corrected for heart rate with the Fridericia formula (QTcF) to determine the risk of cardiac repolarization compared with placebo. Additionally, the PK, safety, and tolerability of a single supratherapeutic (300‐mg) dose of atogepant were evaluated. Moxifloxacin 400 mg was used as the active control and to evaluate assay sensitivity, as moxifloxacin produces a robust and predictable increase in corrected QT (QTc) interval in healthy adults at a time to peak plasma concentration (t_max_) of ≈ 2 hours after dosing.^12^

## Methods

### Study Design

This was a randomized, single‐center, double‐blind, 3‐period, 6‐sequence, single‐dose, crossover, phase 1 trial. Participants were randomly assigned to 1 of 6 intervention sequences (Table S1) to receive 3 interventions across 3 periods: (1) a single oral atogepant 300‐mg supratherapeutic dose (5 × 60‐mg tablets; double‐blinded); (2) a single oral atogepant‐matching placebo (5 tablets; double‐blinded); or (3) a single oral moxifloxacin 400‐mg dose (1 × 400‐mg tablet; open‐label). Participants fasted from 10 hours before through 4 hours after dose administration. Participants received a single dose/intervention on days 1, 8, and 15, separated by a washout period; the maximum duration of the 3 combined periods was 17 days. Follow‐up visits were conducted within 7 days after the last PK sample collection (end‐of‐treatment visit) and at day 45 (± 2 days).

This study was designed, conducted, and analyzed in accordance with the US Food and Drug Administration (FDA) E14 guidance for clinical evaluation of QT/QTc interval prolongation and proarrhythmic potential for non‐antiarrhythmic drugs, and was conducted in accordance with the Declaration of Helsinki and the International Conference on Harmonization E6 guideline for good clinical practice. The study was conducted at PPD Phase I Clinic (Austin, Texas). The study protocol was approved by IntegReview Institutional Review Board (Austin, Texas) before the study was initiated. All participants provided written informed consent before initiation of any study‐specific procedures.

### Participants

Healthy adults 18 to 45 years of age (inclusive) who were nonsmokers and nonusers of nicotine‐containing products (within the previous 2 years), had a supine pulse rate ≥50 and ≤100 beats per minute (bpm), and had a body mass index ≥18.0 and ≤30.0 kg/m^2^ at screening were eligible to participate in the trial.

Exclusion criteria included hypersensitivity to CGRP receptor antagonists or moxifloxacin; any clinical condition that might affect the absorption, distribution, biotransformation, or excretion of atogepant or moxifloxacin; supine systolic blood pressure (BP) ≥140 mm Hg or ≤90 mm Hg or supine diastolic BP ≥90 mm Hg or ≤50 mm Hg; potentially clinically significant electrocardiogram (ECG) results or QT prolongation (QTcF ≥450 milliseconds or uncorrected QT ≥500 milliseconds); history of cardiovascular disease, including cardiac arrhythmia, orthostatic hypotension, coronary artery or valvular disease, or personal or family history of long QT syndrome; clinically significant findings on physical examination, medical history, serum chemistry, hematology, or urinalysis; participation in a clinical investigation requiring multiple blood/plasma collections within 60 days or participation in a blood or plasma donation program within 60 or 30 days, respectively, before dosing; use of hormonal products or St. John's wort within 30 days or concomitant medications (including over‐the‐counter medications) except for select contraceptives within 14 days before dosing; consumption of foods that affect drug metabolizing enzymes and transporters (eg, grapefruit, vegetables of the mustard green family) within 14 days before dosing; consumption of alcohol or caffeine within 72 or 48 hours, respectively, of dosing; or history of alcohol or other substance abuse within the previous 5 years or positive result at screening or day –1 for specific drugs of abuse.

### Intervention Assignment and Blinding

Participants were consecutively assigned identification numbers by the investigator, and a computerized randomization scheme was created by the study sponsor. The randomization scheme was available only to clinic staff who prepared the interventions and who were not involved in any other aspect of the study. The investigator and participants were blinded to interventions with atogepant and placebo. Moxifloxacin was administered open‐label, but the ECG reader was blinded to all study interventions. The bioanalytical laboratory that measured plasma concentrations of study drugs was unblinded to identify study drugs in blood samples.

### Outcomes

The change in QTcF interval from baseline (∆QTcF) following administration of atogepant or placebo was evaluated. For this pharmacodynamic (PD) analysis, the end point was QTcF findings on repeated, digitally recorded, 12‐lead ECGs extracted from Holter monitors. During each of the 3 periods, Holter monitors were used to capture continuous ECGs; ECG data were extracted and analyzed by a core ECG laboratory (eResearch Technology, Inc., Philadelphia, Pennsylvania) according to a prespecified algorithm. Holter ECGs were recorded continuously starting at ≈ 1 hour before dose administration on days 1, 8, and 15 until ≈ 25 hours after dosing. Holter monitor ECG extractions were obtained at 20 minutes, 10 minutes, and immediately before dosing (averaged to use as baseline), and at 0.5, 1, 1.5, 2, 3, 4, 6, 8, 12, and 24 hours after dosing. ECGs were extracted in triplicate within a 5‐minute window around the prespecified ECG nominal time points, and the middle ECG was collected at the nominal time point.

Additional end points included PD variables, PK parameters derived from atogepant and moxifloxacin plasma concentrations, and safety. PD variables included the rate of extreme values for QTcF interval (>450, >480, >500 milliseconds) and QTcF interval change from predose baseline (>30, >60 milliseconds). Blood samples to determine atogepant or moxifloxacin concentrations were collected before dosing and at 0.5, 1, 1.5, 2, 3, 4, 6, 8, 12, and 24 hours after dosing on days 1, 8, and 15. Plasma concentrations of atogepant and moxifloxacin were measured using validated liquid chromatography with tandem mass spectrometry methods as previously described.^13^, ^14^ The lower limit of quantitation for atogepant and moxifloxacin was 10 ng/mL. Safety was assessed by monitoring adverse events, physical examinations, clinical laboratory tests, vital signs, and ECGs.

### Statistical Analysis

The sample size was based on prior clinical experience in QT trials that reported a standard deviation of 9 milliseconds for ΔQTcF from baseline, an average correlation among measurements in the same period of 0.40, and an average correlation among measurements in different periods of 0.15. Sample size estimations were based on the assumptions that the true difference in ΔQTcF from baseline between atogepant and placebo would be 2 milliseconds at prespecified time points of 0.5, 1, 1.5, 2, 3, 4, 6, 8, 12, and 24 hours after dosing. Therefore, a sample size of 48 participants would be needed to complete the study to provide 90% probability that the observed 90% confidence interval (CI) for the true difference in QTcF between atogepant and placebo would fall completely below 10 milliseconds at all time points and an 80% probability of detecting a 5‐millisecond true mean difference between moxifloxacin and placebo for at least 1 time point after adjustment for multiplicity. Thus, assuming a 20% dropout rate, a total sample size of 60 participants was planned for enrollment in the study.

A repeated‐measures model was used to evaluate the effect of atogepant vs placebo and of moxifloxacin vs placebo in ΔQTcF (ΔΔQTcF). The model included predose baseline for the period, predose baseline averaged across periods, intervention, time, and intervention‐by‐time interaction as fixed effects, and participant as a random effect. An unstructured covariance structure was used to model the covariance of the within‐participant values in the same period. The least squares (LS) mean estimate of intervention effect was calculated as well as corresponding 2‐sided 90%CI for each postdose time point. The largest upper limit of the 2‐sided 90%CIs for atogepant vs placebo was compared with the threshold of 10 milliseconds to evaluate the effect of atogepant on QTcF. Assay sensitivity was assessed by determining the time‐matched mean difference between moxifloxacin and placebo in ΔQTcF using the same model that was used in the primary PD analysis of atogepant vs placebo. The largest lower limit of the 2‐sided 90%CI for moxifloxacin vs placebo from the 2‐, 3‐, and 4‐hour time points was compared with the threshold of 5 milliseconds to evaluate the effect of moxifloxacin on QTcF. The Hochberg procedure^15^ was used to control the multiplicity of comparison at multiple time points.

PK parameters that were determined from plasma concentrations of atogepant and moxifloxacin included C_max_, AUC from time 0 to 24 hours and from time 0 to time t, and t_max_. Treatment‐emergent adverse events (TEAEs) were summarized by incidence and coded using the Medical Dictionary for Regulatory Activities version 21.1.

Baseline demographics and safety analyses were conducted using the safety population, which included all participants who received at least 1 dose of study intervention. PD and PK analyses were conducted on the PD population and PK population, respectively, which comprised participants who had evaluable PD or PK data available. PK parameters were analyzed using Phoenix WinNonlin version 8.0 software (Certara LP, Princeton, New Jersey). PK and safety data were analyzed using SAS version 9.4 (SAS Institute, Cary, North Carolina).

## Results

### Participants

A total of 60 participants were enrolled in the study, with 10 participants in each of the 6 intervention sequences. All 60 participants received atogepant, 59 received moxifloxacin, and 59 received placebo. One participant discontinued from the study for a protocol deviation after receiving the first intervention in the sequence (atogepant administered on day 1; participant discontinued on day 7). The mean (standard deviation) age was 33.6 (7.3) years, 32 (53.3%) were women, and 34 (56.7%) were White (Table 1). The safety, PD, and PK populations all included all 60 participants.

**Table 1:** Participant Demographics (Safety Population)

	Placebo (N = 59)	Atogepant 300 mg (N = 60)	Moxifloxacin 400 mg (N = 59)	All Participants (N = 60)
Age, y, mean (SD) [range]	33.5 (7.3) [18–44]	33.6 (7.3) [18–44]	33.5 (7.3) [18–44]	33.6 (7.3) [18–44]
Sex, n (%)				
Female	31 (52.5)	32 (53.3)	31 (52.5)	32 (53.3)
Male	28 (47.5)	28 (46.7)	28 (47.5)	28 (46.7)
Race, n (%)				
White	34 (57.6)	34 (56.7)	34 (57.6)	34 (56.7)
Black/African American	22 (37.3)	23 (38.3)	22 (37.3)	23 (38.3)
Asian	2 (3.4)	2 (3.3)	2 (3.4)	2 (3.3)
Native Hawaiian or Other Pacific Islander	1 (1.7)	1 (1.7)	1 (1.7)	1 (1.7)
Ethnicity, n (%)				
Hispanic	22 (37.3)	22 (36.7)	22 (37.3)	22 (36.7)
Non‐Hispanic	37 (62.7)	38 (63.3)	37 (62.7)	38 (63.3)
Weight, kg, mean (SD)	71.8 (12.2)	71.9 (12.2)	71.8 (12.2)	71.9 (12.2)
Height, cm, mean (SD)	167.0 (8.7)	167.0 (8.7)	167.0 (8.7)	167.0 (8.7)
BMI, kg/m^2^, mean (SD)	25.6 (2.9)	25.7 (2.9)	25.6 (2.9)	25.7 (2.9)

BMI, body mass index; SD, standard deviation.

### Assay Sensitivity

**Figure 1:**
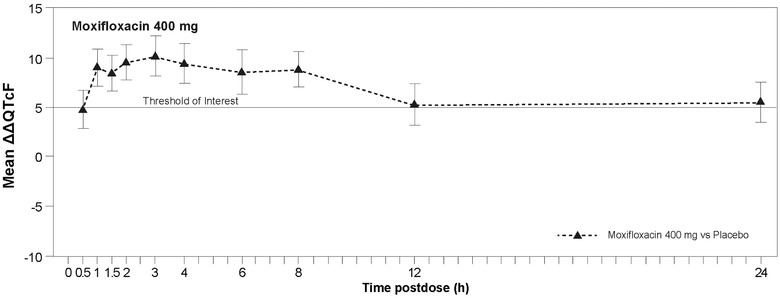
Placebo‐corrected least squares mean (90% confidence interval) change from predose baseline in time‐matched Fridericia‐corrected QT interval (ΔΔQTcF) following moxifloxacin administration (pharmacodynamic population; N = 59).

Assay sensitivity was demonstrated for the measurement of QTcF intervals at the prespecified time points (Figure 1 and Table S2). The lower limit of the 2‐sided 90%CI (LS mean difference) between moxifloxacin and placebo was 9.6 milliseconds (7.7‐11.4) at 2 hours, 10.3 milliseconds (8.2‐12.3) at 3 hours, and 9.5 milliseconds (7.5‐11.4) at 4 hours. The lower limits of the 90%CIs were above the 5‐millisecond threshold at these time points, confirming assay sensitivity.

### Pharmacodynamics

Mean changes in mean heart rate from baseline at 2 hours following atogepant administration were minimal (≤4.2 bpm; Table S3). The robustness of the Fridericia formula for calculating the QTc interval was evaluated by assessing the relationship between QTcF and RR interval. No trend or linear relationship was identified (Figure S1).

**Figure 2:**
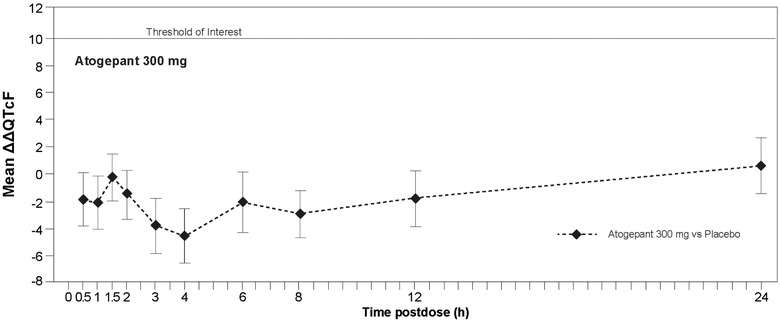
Placebo‐corrected least squares mean (90% confidence interval) change from predose baseline in time‐matched Fridericia‐corrected QT interval (ΔΔQTcF) following atogepant administration (pharmacodynamic population; N = 60).

**Figure 3:**
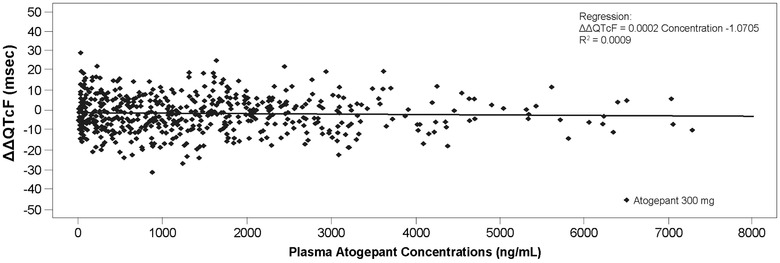
Scatter plot of placebo‐corrected change from baseline intime‐matched Fridericia‐corrected QT interval (ΔΔQTcF) versus plasma atogepant concentration (pharmacodynamic population; N = 60).

Following a single, supratherapeutic oral dose of atogepant 300 mg, the LS mean ΔΔQTcF and upper 2‐sided 90%CIs were lower than the 10‐millisecond threshold at all time points (Figure 2 and Table 2). A maximum ΔΔQTcF of +0.6 milliseconds was observed at 24 hours after atogepant 300‐mg dosing and ΔΔQTcF was <0 at the remaining time points, indicating a potential minimal shortening rather than a prolongation. In contrast, the mean effect of a single dose of 400‐mg moxifloxacin at the prespecified time points was statistically different from placebo, with a lower 2‐sided 90%CI above the 5‐millisecond threshold (Table 2). A scatter plot of the LS mean ΔΔQTcF intervals vs plasma atogepant concentration showed no trends in ΔΔQTcF with increasing plasma atogepant concentration (Figure 3).

**Table 2:** Mean ΔΔQTcF Following a Single Dose of 300‐mg Atogepant or 400‐mg Moxifloxacin (PD Population)

	300‐mg Atogepant‐Placebo	400‐mg Moxifloxacin‐Placebo
Time Point, h	LS Mean Difference (90%CI)	LS Mean Difference (90%CI)
0.5	–1.9 (–3.9 to 0.0)^a^	4.8 (2.8 to 6.8)
1	–2.1 (–4.0 to –0.2)^a^	9.1 (7.2 to 11.1)^b^
1.5	–0.2 (–1.9 to 1.5)^a^	8.5 (6.7 to 10.2)^b^
2	–1.5 (–3.4 to 0.3)^a^	9.6 (7.7 to 11.4)^b^, ^c^
3	–3.8 (–5.9 to –1.8)^a^	10.3 (8.2 to 12.3)^b^, ^c^
4	–4.5 (–6.5 to –2.5)^a^	9.5 (7.5 to 11.4)^b^, ^c^
6	–2.1 (–4.3 to 0.1)^a^	8.6 (6.4 to 10.8)^b^
8	–2.9 (–4.7 to –1.2)^a^	8.9 (7.1 to 10.7)^b^
12	–1.8 (–3.9 to 0.3)^a^	5.3 (3.2 to 7.5)
24	0.6 (–1.4 to 2.6)^a^	5.6 (3.5 to 7.6)

LS, least squares; PD, pharmacodynamic; ΔΔQTcF, placebo‐corrected change from predose baseline in time‐matched Fridericia‐corrected QT interval.

^a^ *P* < .0001; *P* value is based on a test to show that the mean change from predose baseline in QTcF interval for atogepant is <10 msec than that of placebo at the time point.

^b^ *P* < .01; *P* value is based on a test to show that the mean change from predose baseline in QTcF interval for moxifloxacin is >5 msec than that of placebo at the time point.

^c^ *P* < .0001; adjusted *P* value is based on the Hochberg procedure for multiplicity adjustment for assessments at the 2‐, 3‐, and 4‐hour time points.

An extreme value for QTcF interval (outlier; >450 and <480 milliseconds) was reported for only 1 participant at 6 hours after atogepant administration, and for multiple participants at 1 (n = 2), 1.5 (n = 2), 2 (n = 1), and 4 (n = 3) hours after moxifloxacin administration (Table S4). QTcF interval change from baseline >30 milliseconds was reported in 1 participant at 1 hour after moxifloxacin dosing (Table S5).

### Pharmacokinetics

**Figure 4:**
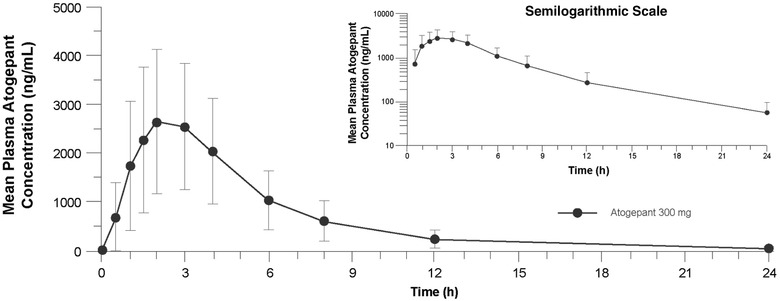
Mean (standard deviation) plasma atogepant concentrations following 300‐mg single‐dose administration (pharmacokinetic population; N = 60) on a linear scale, and semilogarithmic scale (inset).

**Figure 5:**
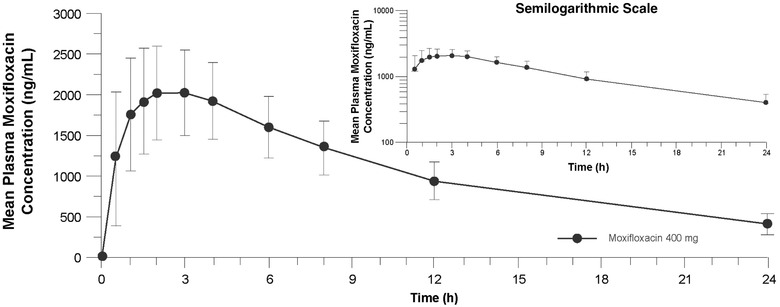
Mean (standard deviation) plasma moxifloxacin concentrations following 400‐mg single‐dose administration (pharmacokinetic population; N = 59) on a linear scale, and semilogarithmic scale (inset).

Atogepant was rapidly absorbed, with a median t_max_ of 2.1 hours. Mean plasma concentration‐time profiles and PK parameter values of atogepant following single‐dose administration are shown in Figure 4 and Table 3, respectively, and were consistent with results from other atogepant PK studies of varying atogepant doses. Mean plasma concentration‐time profiles and PK parameter values for moxifloxacin following single‐dose administration of moxifloxacin 400 mg are shown in Figure 5 and Table 3, respectively, and were consistent with ranges from previously published studies.^12^, ^16^, ^17^

**Table 3:** PK Parameter Values Following Single‐Dose Oral Administration of Atogepant 300 mg and Moxifloxacin 400 mg (PK Population)

PK Parameter, Mean (SD)	Atogepant 300 mg (N = 60)	Moxifloxacin 400 mg (N = 59)
C_max_, ng/mL	3197 (1531)	2301 (573)
AUC_0‐24_, ng • h/mL	16 607 (8242)	25 752 (6007)
AUC_0‐t_, ng • h/mL	16 640 (8254)	25 931 (6037)
t_max_, h^a^	2.1 (1.1‐6.1)	2.1 (0.6‐6.1)

AUC_0‐24_, area under the concentration‐time curve from time 0 to 24 h; AUC_0‐t_, area under the concentration‐time curve from time 0 to time t; C_max_, peak plasma concentration; PK, pharmacokinetic; SD, standard deviation; t_max_, time to peak plasma concentration.

^a^ Median (range).

### Safety

There were no treatment‐emergent serious adverse events, deaths, or TEAEs leading to intervention discontinuation during the study. All TEAEs were mild or moderate in severity, and none were considered by the investigator to be related to any study intervention. Fewer participants had a TEAE following dosing with atogepant (1.7%) than with placebo (5.1%) or moxifloxacin (13.6%) (Table 4). The most commonly reported TEAEs were nausea (n = 3), headache (n = 3), and somnolence (n = 2) (Table S6).

**Table 4:** Summary of Adverse Events by Treatment (Safety Population)

Participants With Event, n (%)	Placebo (N = 59)	Atogepant 300 mg (N = 60)	Moxifloxacin 400 mg (N = 59)
Any TEAE	3 (5.1)	1 (1.7)	8 (13.6)
Serious TEAE	0	0	0
Treatment‐related TEAE	0	0	0
TEAE leading to discontinuation	0	0	0

TEAE, treatment‐emergent adverse event.

Abnormal clinical laboratory values were reported for 3 participants: absolute neutrophil count <0.8 × 10^9^/L was observed in 1 participant in sequence 1 (atogepant‐placebo‐moxifloxacin) at the end of treatment and in 1 participant in sequence 5 (placebo‐moxifloxacin‐atogepant) at the end of treatment; and 1 participant had an albumin value <0.9 g/L following dosing with moxifloxacin. None of the abnormal laboratory values were considered by the investigator to be clinically relevant, and none were reported as a TEAE.

No participant had laboratory values meeting the criteria for a potential Hy's Law case (alanine aminotransferase or aspartate aminotransferase ≥3 times the upper limit of normal [ULN], total bilirubin ≥2 times the ULN, and alkaline phosphatase <2 times the ULN), and no participant had significantly elevated liver laboratory parameters.

Abnormal vital signs were recorded in 3 participants: 1 participant had a pulse rate ≤50 bpm and a decrease ≥15 bpm from baseline at the end of treatment; 1 participant had a pulse rate ≤50 bpm and a decrease ≥15 bpm from baseline after dosing with placebo; and 1 participant had a diastolic BP ≤50 mm Hg and a decrease ≥15 mm Hg following dosing with moxifloxacin. None of the abnormal vital signs were reported as a TEAE. No participant had a clinically significant ECG abnormality.

## Discussion

The findings of this study, which was conducted in accordance with the US FDA E14 guidance for clinical evaluation of QT/QTc interval prolongation, suggest that atogepant has no clinically relevant effect on cardiac repolarization.

The sensitivity of the assay was demonstrated by a statistically significant prolongation of the baseline‐corrected QTcF interval of moxifloxacin 400 mg vs placebo, as the lower limits of the 2‐sided 90%CIs were above the 5‐millisecond threshold at the prespecified 2‐, 3‐, and 4‐hour time points. Moxifloxacin PK parameter values observed in this study were consistent with other studies, which reported mean AUC values of 20 300 to 44 600 ng • h/mL and mean C_max_ values of 1620 to 3800 ng/mL.^12^, ^16^, ^17^

Systemic exposure to supratherapeutic levels of atogepant observed in this study was as expected based on dose‐proportional increases in AUC reported for lower doses.^13^ Single‐dose and multiple‐dose PK data suggested that the AUC and C_max_ of atogepant increased in a roughly dose‐proportional manner from 40 to 170 mg, and atogepant does not undergo significant accumulation at steady‐state regardless of dose. Based on systemic exposure of atogepant observed at a 300‐mg single dose in a small cohort of healthy participants before thorough QT evaluation, a single supratherapeutic dose of 300‐mg atogepant was expected to provide AUC and C_max_ values ≈ 5‐fold greater than the highest clinical dose of 60 mg. Notably, a supratherapeutic dose of atogepant had no impact on cardiac repolarization in healthy adult participants, as shown by the absence of any ΔΔQTcF value attaining the 10‐millisecond threshold for the upper limit of the 2‐sided 90%CIs. In healthy participants, the incidence of TEAEs with atogepant was low, and no clinically meaningful changes from baseline were reported for vital signs, clinical laboratory, or ECG parameters.

In a phase 2b/3 clinical trial, all tested doses of atogepant (10 mg once daily, 30 mg once daily, 60 mg once daily, 30 mg twice daily, and 60 mg twice daily) were superior to placebo in reducing mean monthly migraine days across 12 weeks of treatment.^10^ Nausea was the only treatment‐related TEAE occurring in at least 5% of participants in multiple‐dose atogepant groups. During the trial, no treatment‐related serious adverse events were reported with atogepant. After daily dosing for 12 weeks, there were no clinically relevant changes in laboratory parameters, vital signs, and safety ECGs, nor were there any potential Hy's Law cases or concerns about drug‐induced liver injury.

Although this type of study was conducted in accordance with FDA guidance to examine QT/QTc interval prolongation during drug development, a potential limitation of the study is that only a single supratherapeutic dose was evaluated. In a phase 3 clinical trial, people with migraine received daily doses of atogepant 10, 30, or 60 mg for 12 weeks.^11^ While only a single dose of atogepant 300 mg was evaluated in this study, no relationship between atogepant concentrations and ΔΔQTcF was found. Additionally, the present study did not evaluate the impact of multiple‐day administration or change in QT/QTc interval beyond 24 hours after dosing. Given the minimal accumulation of atogepant with once‐daily dosing and the observation that a single dose of 300‐mg atogepant resulted in systemic exposures well above those observed at therapeutic doses with single‐dose administration, multiple‐dose administration is unlikely to affect the outcome of this study. Another potential limitation is that the current study included only healthy adult participants, which is not the target population to receive atogepant in clinical practice.

## Conclusion

In this thorough QT study, a single supratherapeutic dose of atogepant did not significantly prolong QT intervals in healthy participants based on an assay with sensitivity confirmed by results observed with moxifloxacin. A single supratherapeutic dose of atogepant was safe in healthy adult participants.

## Conflicts of Interest

R.B., B.M., M.B., D.M., and L.B. are employees of AbbVie, and may hold AbbVie stock. L.Y. and A.P. were employees of AbbVie at the time of this study, and may hold AbbVie stock.

## Funding

This study was funded by Allergan (before its acquisition by AbbVie).

## Author Contributions

RB, BM, LY, and AP designed the research, RB, BM, MB, DM, and LB performed the research, and RB, BM, LB, and LY analyzed and interpreted the data. All authors wrote the manuscript, revised the manuscript for intellectual content, and gave final approval of the completed manuscript.

## Supporting information

Additional supplemental information can be found by clicking the Supplements link in the PDF toolbar or the Supplemental Information section at the end of the web‐based version of this article.

Supplementary information

## Data Availability

AbbVie is committed to responsible data sharing regarding the clinical trials we sponsor. This includes access to anonymized, individual and trial‐level data (analysis data sets), as well as other information (eg, protocols and Clinical Study Reports), as long as the trials are not part of an ongoing or planned regulatory submission. This includes requests for clinical trial data for unlicensed products and indications. This clinical trial data can be requested by any qualified researchers who engage in rigorous, independent scientific research, and will be provided following review and approval of a research proposal and Statistical Analysis Plan (SAP) and execution of a Data Sharing Agreement (DSA). Data requests can be submitted at any time and the data will be accessible for 12 months, with possible extensions considered. For more information on the process, or to submit a request, visit the following link: https://www.abbvie.com/our-science/clinical-trials/clinical-trials-data-and-information-sharing/data-and-information-sharing-withqualified-researchers.html.
