# Supplementary material for: A Single Supratherapeutic Dose of Atogepant Does Not Affect Cardiac Repolarization in Healthy Adults: Results From a Randomized, Single‐Dose, Phase 1 Crossover Trial
Source: Clin Pharmacol Drug Dev. 2021 May 4;10(9):1099–107. doi: 10.1002/cpdd.940 (PMC8453716; doi:10.1002/cpdd.940)

**SUPPLEMENTAL MATERIALS**

**A Single Supratherapeutic Dose of Atogepant Does Not Affect Cardiac Repolarization in Healthy Adults: Results From a Randomized, Single-Dose, Phase 1 Crossover Trial**

Ramesh Boinpally, Brian McNamee, Li Yao, Matthew Butler, Danielle McGeeney,
Lisa Borbridge, Antonia Periclou

**Table S1.** Study Interventions.

**Table S2.** Mean ΔΔQTcF Interval Over Time Following a Single Dose of 400 mg Moxifloxacin (Pharmacodynamic Population).

**Table S3.** Mean Change From Baseline in Mean Heart Rate (beats/min) at 2 and 24 Hours Postdose.

**Table S4.** Participants With Extreme Values for QTcF Interval (Pharmacodynamic Population).

**Table S5.** Participants With Extreme QTcF Interval Change From Baseline (Pharmacodynamic Population).

**Table S6.** Treatment-Emergent Adverse Events by Treatment, System Organ Class, and Preferred Term (Safety Population).

**Figure S1.** Scatter Plot of QTcF Versus RR Interval

**Table S1.** Study Interventions.

| **Sequence** | **Period 1** | **Period 2** | **Period 3** |
| --- | --- | --- | --- |
| 1 | A | B | C |
| 2 | B | A | C |
| 3 | C | B | A |
| 4 | A | C | B |
| 5 | B | C | A |
| 6 | C | A | B |

Intervention A: Single oral atogepant 300 mg supratherapeutic dose (5 × 60 mg tablets).

Intervention B: Single oral atogepant-matching placebo (5 tablets).

Intervention C: Single oral moxifloxacin 400 mg dose (1 × 400 mg tablet).

**Table S2.** Mean ΔΔQTcF Interval Over Time Following a Single Dose of 400 mg Moxifloxacin (Pharmacodynamic Population).

| **Time Point, h After Dosing** | **Moxifloxacin-Placebo**  **LS Mean Difference (90% CI)** |
| --- | --- |
| 0.5 | 4.8 (2.8, 6.8) |
| 1 | 9.1 (7.2, 11.1) |
| 1.5 | 8.5 (6.7, 10.2) |
| 2 | 9.6 (7.7, 11.4)^a^ |
| 3 | 10.3 (8.2, 12.3)^a^ |
| 4 | 9.5 (7.5, 11.4)^a^ |
| 6 | 8.6 (6.4, 10.8) |
| 8 | 8.9 (7.1, 10.7) |
| 12 | 5.3 (3.2, 7.5) |
| 24 | 5.6 (3.5, 7.6) |

^a^ *P*≤0.001 based on a test to show that the mean change from predose baseline in QTcF interval for moxifloxacin was >5 msec than that of placebo, and adjusted *P*≤0.001 based on the Hochberg procedure for multiplicity adjustment for assessments at the 2-, 3-, and 4-hour timepoints.

LS, least squares.

**Table S3.** Mean Change From Baseline in Mean Heart Rate (beats/min) at 2 and 24 Hours Postdose

| **Mean Change From Baseline (SD)** | **Placebo (N=59)** | **Atogepant 300 mg (N=60)** | **Moxifloxacin 400 mg (N=59)** |
| --- | --- | --- | --- |
| **2 hours postdose** | -1.2 (4.1) | -4.2 (4.4) | -0.8 (5.0) |
| **24 hours postdose** | 0.9 (4.6) | -0.1 (6.2) | 0.2 (6.4) |

**Table S4.** Participants With Extreme Values for QTcF Interval (Pharmacodynamic Population).

| **Time After Dosing**  **QTcF Interval** | **Placebo (N=59) n/N1 (%)** | **Atogepant 300 mg (N=60) n/N1 (%)** | **Moxifloxacin 400 mg (N=59) n/N1 (%)** |
| --- | --- | --- | --- |
| Baseline |  |  |  |
| >450 msec | 0/59 (0) | 0/60 (0) | 0/59 (0) |
| >480 msec | 0/59 (0) | 0/60 (0) | 0/59 (0) |
| >500 msec | 0/59 (0) | 0/60 (0) | 0/59 (0) |
| 0.5 hr |  |  |  |
| >450 msec | 0/58 (0) | 0/60 (0) | 0/59 (0) |
| >480 msec | 0/58 (0) | 0/60 (0) | 0/59 (0) |
| >500 msec | 0/58 (0) | 0/60 (0) | 0/59 (0) |
| 1 hr |  |  |  |
| >450 msec | 0/58 (0) | 0/60 (0) | 2/59 (3.4) |
| >480 msec | 0/58 (0) | 0/60 (0) | 0/59 (0) |
| >500 msec | 0/58 (0) | 0/60 (0) | 0/59 (0) |
| 1.5 hrs |  |  |  |
| >450 msec | 0/58 (0) | 0/60 (0) | 2/59 (3.4) |
| >480 msec | 0/58 (0) | 0/60 (0) | 0/59 (0) |
| >500 msec | 0/58 (0) | 0/60 (0) | 0/59 (0) |
| 2 hrs |  |  |  |
| >450 msec | 0/58 (0) | 0/60 (0) | 1/59 (1.7) |
| >480 msec | 0/58 (0) | 0/60 (0) | 0/59 (0) |
| >500 msec | 0/58 (0) | 0/60 (0) | 0/59 (0) |
| 3 hrs |  |  |  |
| >450 msec | 0/59 (0) | 0/60 (0) | 0/59 (0) |
| >480 msec | 0/59 (0) | 0/60 (0) | 0/59 (0) |
| >500 msec | 0/59 (0) | 0/60 (0) | 0/59 (0) |
| 4 hrs |  |  |  |
| >450 msec | 0/59 (0) | 0/60 (0) | 3/59 (5.1) |
| >480 msec | 0/59 (0) | 0/60 (0) | 0/59 (0) |
| >500 msec | 0/59 (0) | 0/60 (0) | 0/59 (0) |
| 6 hrs |  |  |  |
| >450 msec | 0/58 (0) | 1/60 (1.7) | 0/59 (0) |
| >480 msec | 0/58 (0) | 0/60 (0) | 0/59 (0) |
| >500 msec | 0/58 (0) | 0/60 (0) | 0/59 (0) |
| 8 hrs |  |  |  |
| >450 msec | 0/59 (0) | 0/60 (0) | 0/58 (0) |
| >480 msec | 0/59 (0) | 0/60 (0) | 0/58 (0) |
| >500 msec | 0/59 (0) | 0/60 (0) | 0/58 (0) |
| 12 hrs |  |  |  |
| >450 msec | 0/59 (0) | 0/60 (0) | 0/57 (0) |
| >480 msec | 0/59 (0) | 0/60 (0) | 0/57 (0) |
| >500 msec | 0/59 (0) | 0/60 (0) | 0/57 (0) |
| 24 hrs |  |  |  |
| >450 msec | 0/59 (0) | 0/60 (0) | 0/57 (0) |
| >480 msec | 0/59 (0) | 0/60 (0) | 0/57 (0) |
| >500 msec | 0/59 (0) | 0/60 (0) | 0/57 (0) |

n, number of participants in category; N1, number of participants with available data.

**Table S5.** Participants With Extreme QTcF Interval Change From Baseline (Pharmacodynamic Population).

| **Time After Dosing**  **QTcF Interval Change From Baseline** | **Placebo (N=59) n/N1 (%)** | **Atogepant 300 mg (N=60) n/N1 (%)** | **Moxifloxacin 400 mg (N=59) n/N1 (%)** |
| --- | --- | --- | --- |
| 0.5 hr |  |  |  |
| >30 msec | 0/58 (0) | 0/60 (0) | 0/59 (0) |
| >60 msec | 0/58 (0) | 0/60 (0) | 0/59 (0) |
| 1 hr |  |  |  |
| >30 msec | 0/58 (0) | 0/60 (0) | 1/59 (1.7) |
| >60 msec | 0/58 (0) | 0/60 (0) | 0/59 (0) |
| 1.5 hrs |  |  |  |
| >30 msec | 0/58 (0) | 0/60 (0) | 0/59 (0) |
| >60 msec | 0/58 (0) | 0/60 (0) | 0/59 (0) |
| 2 hrs |  |  |  |
| >30 msec | 0/58 (0) | 0/60 (0) | 0/59 (0) |
| >60 msec | 0/58 (0) | 0/60 (0) | 0/59 (0) |
| 3 hrs |  |  |  |
| >30 msec | 0/59 (0) | 0/60 (0) | 0/59 (0) |
| >60 msec | 0/59 (0) | 0/60 (0) | 0/59 (0) |
| 4 hrs |  |  |  |
| >30 msec | 0/59 (0) | 0/60 (0) | 0/59 (0) |
| >60 msec | 0/59 (0) | 0/60 (0) | 0/59 (0) |
| 6 hrs |  |  |  |
| >30 msec | 0/58 (0) | 0/60 (0) | 0/59 (0) |
| >60 msec | 0/58 (0) | 0/60 (0) | 0/59 (0) |
| 8 hrs |  |  |  |
| >30 msec | 0/59 (0) | 0/60 (0) | 0/58 (0) |
| >60 msec | 0/59 (0) | 0/60 (0) | 0/58 (0) |
| 12 hrs |  |  |  |
| >30 msec | 0/59 (0) | 0/60 (0) | 0/57 (0) |
| >60 msec | 0/59 (0) | 0/60 (0) | 0/57 (0) |
| 24 hrs |  |  |  |
| >30 msec | 0/58 (0) | 0/60 (0) | 0/56 (0) |
| >60 msec | 0/58 (0) | 0/60 (0) | 0/56 (0) |

n, number of participants in category; N1, number of participants with available data.

**Table S6.** Treatment-Emergent Adverse Events by Treatment, System Organ Class, and Preferred Term (Safety Population).

| **Primary System Organ Class Preferred Term, n (%)** | **Placebo (N=59)** | **Atogepant 300 mg (N=60)** | **Moxifloxacin 400 mg (N=59)** |
| --- | --- | --- | --- |
| Participant with ≥1 TEAE | 3 (5.1) | 1 (1.7) | 8 (13.6) |
| **Eye disorders** | **1 (1.7)** | **0** | **0** |
| Photophobia | 1 (1.7) | 0 | 0 |
| **Gastrointestinal disorders** | **1 (1.7)** | **1 (1.7)** | **4 (6.8)** |
| Nausea | 0 | 0 | 3 (5.1) |
| Abdominal distension | 0 | 0 | 1 (1.7) |
| Constipation | 1 (1.7) | 0 | 0 |
| Diarrhea | 0 | 1 (1.7) | 0 |
| Vomiting | 0 | 0 | 1 (1.7) |
| **General disorders and administration site conditions** | **1 (1.7)** | **0** | **0** |
| Asthenia | 1 (1.7) | 0 | 0 |
| **Injury, poisoning, and procedural complications** | **1 (1.7)** | **0** | **0** |
| Arthropod bite | 1 (1.7) | 0 | 0 |
| **Nervous system disorders** | **0** | **0** | **4 (6.8)** |
| Headache | 0 | 0 | 3 (5.1) |
| Somnolence | 0 | 0 | 2 (3.4) |
| Dizziness | 0 | 0 | 1 (1.7) |
| **Renal and urinary disorders** | **0** | **0** | **1 (1.7)** |
| Dysuria | 0 | 0 | 1 (1.7) |
| Pollakiuria | 0 | 0 | 1 (1.7) |
| **Respiratory, thoracic, and mediastinal disorders** | **0** | **0** | **1 (1.7)** |
| Epistaxis | 0 | 0 | 1 (1.7) |

TEAE, treatment-emergent adverse event.

**Figure S1.** Scatter Plot of QTcF Versus RR Interval


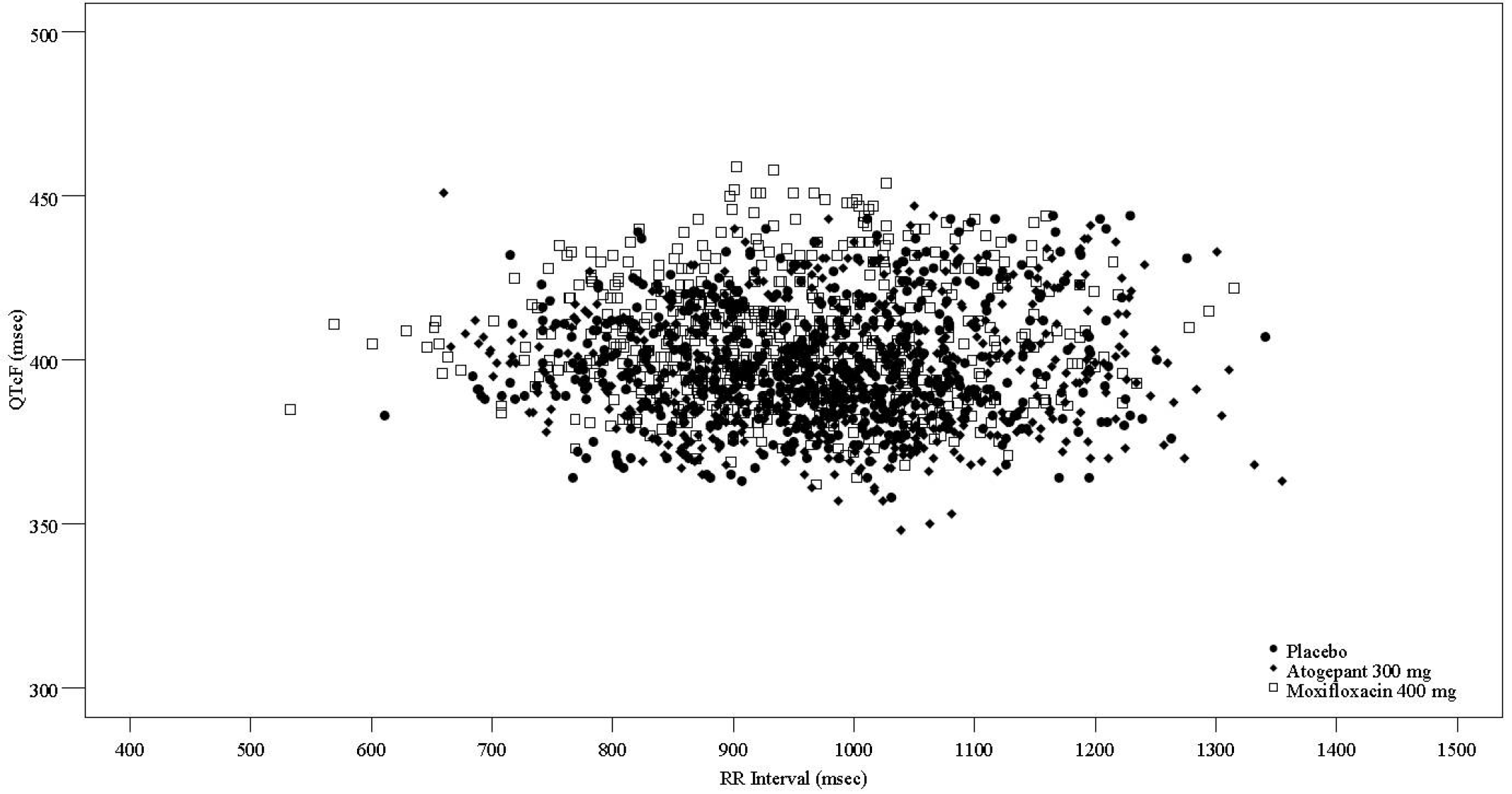

Supplement: Supplementary file 1 — Additional supplemental information can be found by clicking the Supplements link in the PDF toolbar or the Supplemental Information section at the end of the web‐based version of this article. [file CPDD-10-1099-s001.docx]
